# First report of an *Escherichia coli* ST131 clinical isolate co-harboring *bla*_KPC-2_ and *bla*_NDM-13_ on an IncB/O/K/Z plasmid in China

**DOI:** 10.1128/spectrum.00528-25

**Published:** 2025-12-18

**Authors:** Yi-Yu Lyu, Jie-Hao Tai, Cui-Ying Guo, Yin-Yin Zhang, Yao Chen, Qiang Zhou, Wen-Wen Chu, Yi-Le Wu

**Affiliations:** 1 Department of Clinical Laboratory, the Second Affiliated Hospital of Anhui Medical University, Hefei, Anhui, China; 2 Department of Hospital Infection Prevention and Control, the First People’s Hospital of Bengbu, Bengbu, Anhui, China; 3 Department of Clinical Laboratory, the First People’s Hospital of Bengbu, Bengbu, Anhui, China; 4 Department of Critical Care Medicine, the First People’s Hospital of Bengbu, Bengbu, Anhui, China; 5 Department of Hospital Infection Prevention and Control, the Second Affiliated Hospital of Anhui Medical University, Hefei, Anhui, China; Beijing Institute of Genomics, Chinese Academy of Sciences, Beijing, China

**Keywords:** *Escherichia coli*, *bla*_KPC-2_, *bla*_NDM-13_, IncB/O/K/Z, IS*1294*

## Abstract

In carbapenem-resistant *Enterobacteriaceae*, the co-occurrence of carbapenem resistance genes poses a significant threat to global public health. This study investigated the phenotypic and genotypic characteristics of a clinical carbapenem-resistant *Escherichia coli* strain B5, which harbors both *bla*_KPC-2_ and *bla*_NDM-13_. Antimicrobial susceptibility testing and plasmid conjugation assays were performed on isolate B5, using *E. coli* J53 (a standard recipient strain resistant to sodium azide) as the recipient, whereas passaging experiments and growth rate determination were conducted on J53 (pB5-KPC-NDM). Genetic characteristics of B5 were analyzed via whole-genome sequencing (WGS). B5 exhibits an extensive multidrug resistance phenotype, with susceptibility only to tigecycline and colistin. WGS revealed that B5 belongs to ST131, carries 11 plasmids, and co-harbors *bla*_KPC-2_ and *bla*_NDM-13_ on the IncB/O/K/Z plasmid pB5-KPC-NDM. This plasmid also exhibited considerable stability in J53 (pB5-KPC-NDM), with a retention rate of 74% (37/50) after 10 days of serial passage in antibiotic-free medium. Compared with the recipient strain J53, J53 (pB5-KPC-NDM) imposed a low fitness cost. Additionally, WGS further identified multiple additional resistance genes on pB5-KPC-NDM. Comparative analysis showed that *bla*_KPC-2_ resides within Tn*6296* derivatives and *bla*_NDM-13_ within Tn*125* derivatives on pB5-KPC-NDM, featuring both conserved and unique flanking contexts. Core structures potentially enabling horizontal transfer were identified: ∆Tn*6376-bla*_KPC-2_-∆IS*Kpn6-korC-klcA-∆repB*-∆Tn*1722*-5’ for *bla*_KPC-2_ and IS*1294*-∆IS*Aba125-bla*_NDM-13_-*ble*_MBL_-*trpF-nagA* for *bla*_NDM-13_. Notably, IS*1294* (IS*91* family), replaces IS*Aba125*, is likely to mobilize *bla*_NDM-13_. In conclusion, the pB5-KPC-NDM plasmid poses a severe threat due to its extensive resistance profile, high transferability, and low fitness cost, urging immediate intervention to prevent its dissemination.

**IMPORTANCE:**

Antimicrobial resistance has become a serious global public health concern, severely limiting therapeutic options. The global proliferation of carbapenem-resistant *Enterobacteriaceae*, driven by plasmid-mediated horizontal gene transfer of carbapenemase-encoding elements, constitutes a critical antimicrobial resistance crisis. This study provides the first evidence of *bla*_KPC-2_ and *bla*_NDM-13_ co-occurring on an IncB/O/K/Z plasmid (pB5-KPC-NDM), as well as the first detection of these genes in a clinical *Escherichia coli* isolate (B5). Phenotypic and genotypic analyses demonstrate efficient horizontal transfer capacity and stability across bacterial generations of pB5-KPC-NDM, facilitating the spreading of multidrug resistance. This dual carbapenemase co-localization represents a pivotal escalation in the dissemination potential of resistance and consequently heightens the threat of its spread worldwide. These findings emphasize the critical need for enhanced genomic surveillance programs and the implementation of stringent infection control measures to mitigate the global dissemination of such multidrug-resistant plasmids carrying high-risk carbapenemase variants.

## INTRODUCTION

Antimicrobial resistance has emerged as a critical global public health threat, leading to significant limitations in therapeutic options (1, 2). Carbapenems are the last resort antibiotics for the treatment of multidrug-resistant *Enterobacteriaceae* infections (3). With the extensive use of carbapenems, carbapenem-resistant *Enterobacteriaceae* (CRE) have emerged as a challenging issue and garnered widespread attention (4). *Escherichia coli* is one of the most prevalent *Enterobacteriaceae* in both hospital and community environments, capable of causing various serious infections (5). According to recent data from the 2023 China Antimicrobial Resistance Surveillance System, *E. coli* has become the first and most commonly isolated bacterium in clinics in China, and the resistance rate to carbapenems was 1.7% (6). It causes numerous adverse health effects on patients and imposes a considerable societal burden (3, 7). The primary mechanism of drug resistance gene transmission in CRE is due to the horizontal transfer of mobile genetic elements on plasmids that encode enzymes responsible for hydrolyzing carbapenems and other *β*-lactam agents. Notably, *Klebsiella pneumoniae* carbapenemase (KPC) and New Delhi metallo-*β*-lactamase (NDM) are the most commonly represented carbapenemases found in both inpatient and environmental CRE samples (3).

KPC is classified as class A according to the Ambler classification system and predominantly occurs in carbapenem-resistant *K. pneumoniae* (CRKP) (8) and is widely distributed worldwide (3). Currently, there are more than 150 known *bla*_KPC_ subtypes, with the main novel *bla*_KPC_ variety identified as a mutation of *bla*_KPC-2_ (8). It has been reported that the *bla*_KPC_ gene can be carried by various plasmids, including IncFII, IncI2, IncX, IncA/C, IncN, and IncR (8–10). NDM, which belongs to class B *β*-lactamase, has at least 48 variants listed in the NCBI database. The *bla*_NDM-13_ is a variant characterized by two amino acid substitutions (D95N and M154L) compared to *bla*_NDM-1_, which results in increased hydrolytic activity against cefotaxime (11). The *bla*_NDM_ is primarily carried by plasmids, including IncHI1, IncHI2, IncHI3, IncX3, IncX1, and IncN (12). *K. pneumoniae* and *E. coli* are significant carriers of the *bla*_NDM_ (12). Recently, the coexistence of *bla*_KPC_ and *bla*_NDM_ in bacteria, including *K. pneumoniae* and *E. coli*, has garnered more attention (13, 14).

This study provides the first evidence of *bla*_KPC-2_ and *bla*_NDM-13_ co-occurring on an IncB/O/K/Z plasmid, as well as the first detection of these genes in a clinical *E. coli* isolate (B5). A series of experiments and whole-genome sequencing (WGS) were employed to analyze the phenotypic and genotypic characteristics of the B5, highlighting the urgent need for surveillance of the potential widespread dissemination of such a concerning isolate and its mobile genetic elements on plasmids in the future.

## MATERIALS AND METHODS

### Bacterial isolate

During a 1-year multicenter CRE surveillance study involving 10 intensive care units (ICUs) from July 2023 to June 2024 in Anhui Province, China, an *E. coli* isolate (designated B5) co-harboring *bla*_KPC-2_ and *bla*_NDM-13_ was identified. The isolate was obtained from a rectal swab specimen of a 77-year-old female patient during active CRE screening upon admission to the ICU at Bengbu First People’s Hospital, Bengbu City, Anhui Province, China, on 25 July 2023. The patient presented with pulmonary infection symptoms following transfer from another medical facility, with a documented history of recent hospitalization and mechanical ventilation. Subsequent phenotypic and genomic analyses were conducted to characterize isolate B5.

### Species identification and antimicrobial susceptibility testing

Species identification was performed using the Microflex LT automated microbial identification system (Bruker Daltonics, Germany). Antimicrobial susceptibility testing was performed using the Vitek2 Automated System (BioMérieux, France). Minimum inhibitory concentrations (MICs) for 14 antimicrobial agents, including *β*-lactam/*β*-lactamase inhibitor combinations (amoxicillin-clavulanate, piperacillin-tazobactam), cephalosporins (cefuroxime, cefoxitin, ceftazidime, ceftriaxone, and cefepime), carbapenems (ertapenem, imipenem, meropenem), amikacin, and levofloxacin, were assessed via broth microdilution following CLSI guidelines (15). EUCAST MIC breakpoints were applied for tigecycline and colistin interpretation (16). *E. coli* ATCC 25922 served as the quality control strain.

### Verification of the transferability of the plasmid carrying *bla*_KPC-2_ and *bla*_NDM-13_

A plasmid conjugation experiment was conducted to assess the transferability of the plasmid harboring *bla*_KPC-2_ and *bla*_NDM-13_, utilizing B5 as the donor isolate and sodium azide-resistant *E. coli* J53 as the recipient isolate. Donor and recipient isolates were, respectively, inoculated into fresh Luria–Bertani (LB) broth (Sangon Biotech, Shanghai, China) to prepare a 1.0 McFarland turbidity standard suspension (3 × 10^8^ CFU/mL). Then, they were mixed at a 1:2 donor-to-recipient ratio and incubated on LB agar plates at 37°C for 12 to 24 h. J53 (pB5-KPC-NDM) colonies were selected on Mueller-Hinton (MH) agar plates (Oxoid, Hampshire, UK) containing sodium azide (296 µg/mL) and meropenem (10 µg/mL). Target gene segments of *bla*_KPC-2_ and *bla*_NDM-13_ in J53 (pB5-KPC-NDM) isolates were amplified by polymerase chain reaction (PCR), and the PCR results were then subjected to agarose gel electrophoresis. The primers used in the PCR analysis were *bla*_KPC-2_ (5′-GTATCGCCGTCTAGTTCTGC-3′, 5′-GGTCGTGTTTCCCTTTAGCC-3′), *bla*_NDM-13_ (5′-ATGGAATTGCCCAATATTATGCAC-3′, 5′-TCAGCGCAGCTTGTCGGC-3′), and enterobacterial repetitive intergenic consensus (ERIC) (5′-AAGTAAGTGACTGGGGTGAGCG-3′, 5′-ATGTAAGCTCCTGGGGATTCAC-3′). Electrophoresis was performed with the BG-Power600i System (Boygene, Beijing, China) using 1.0% agarose in Tris Acetate-EDTA buffer at 100 V. The gel electrophoresis profiles were analyzed using Tanon-1600 (Tanon, Shanghai, China).

### Stability of the plasmid carrying *bla*_KPC-2_ and *bla*_NDM-13_

Plasmid retention in J53 (pB5-KPC-NDM) was evaluated through serial passaging in antibiotic-free LB broth. Three single colonies were inoculated into 2 mL LB broth; cultures were diluted 1:1,000 daily in fresh medium and passaged for 10 days. Every fifth day, cultures were serially diluted in PBS and plated on MH agar without antibiotics. Fifty colonies per time point were screened by PCR for *bla*_KPC-2_ and *bla*_NDM-13_. Plasmid retention frequency was calculated as (positive clones/50) × 100%.

### Determination of growth rate

Fitness costs associated with plasmid carriage were assessed by comparing the growth curves of J53 (pB5-KPC-NDM) and recipient J53. Overnight cultures were diluted 1:100 in LB broth, aliquoted into 96-well plates, and incubated with shaking at 37°C. Optical density (OD_600_) was measured hourly for 12 h. Growth curves were analyzed via one-way ANOVA (GraphPad Prism v10.0), with *P* <0.05 considered statistically significant.

### Whole-genome sequencing and analysis

Next-generation sequencing was utilized to sequence and assemble the genome. Genomic DNA was extracted using a plant genomic DNA extraction kit (Tiangen, DP305). A library was prepared and sequenced on an Illumina NovaSeq platform (Illumina Inc., San Diego, CA, USA), generating 150 bp paired-end reads. Quality-controlled reads were assembled *de novo* using Unicycler v0.5.0 (https://github.com/rrwick/Unicycler). The resulting assemblies were integrated to produce a complete sequence. Gene prediction was conducted using Prokka v1.12 software (https://github.com/tseemann/prokka).

Third-generation sequencing was employed for genome sequencing and assembly to further investigate the genetic background of plasmids in isolate B5 and J53 (pB5-KPC-NDM), elucidating their formation pathways. Genomic DNA was extracted using a modified cetyltrimethyl ammonium bromide method. Sequencing was performed by Personal Biotechnology Company (Shanghai, China) using both the Nanopore PromethION 48 platform and the Illumina NovaSeq platform. The resulting data were assembled using Flye v2.9.1 (https://github.com/fenderglass/Flye) and Unicycler v0.5.0. The assemblies were then integrated to generate a complete sequence, which was further polished using Pilon v1.24 software (https://github.com/broadinstitute/pilon?tab=readme-ov-file) to achieve the final high-quality genome sequence.

### Genome component prediction and analysis

Pair-wise average nucleotide identity (ANI) values were calculated using FastANI, with organisms belonging to the same species typically showing ≥95% ANI among themselves (17). Multilocus sequence typing (MLST), antimicrobial resistance genes, and plasmid replicons were identified using the Center for Genomic Epidemiology tools (MLST 2.1, ResFinder 4.1, and PlasmidFinder 1.3 at http://www.genomicepidemiology.org/). Comparative analysis of the IncB/O/K/Z plasmid against reference sequences (GenBank accession no. CP103407, CP168663, CP123269, X61367, FJ628167, JN872328, KX094555) was performed via BLASTn v2.4.0 (https://blast.ncbi.nlm.nih.gov/Blast.cgi) and The Transposon Registry (https://transposon.lstmed.ac.uk/tn-registry). Transposon and insertion sequence (IS) elements were scanned using the ISFinder database (https://www-is.biotoul.fr/index.php). Genomic maps were generated using Proksee (https://proksee.ca/), and linear plasmid comparisons were visualized with Easyfig 2.2.5 (https://mjsull.github.io/Easyfig/files.html).

## RESULTS

### Antimicrobial susceptibility test

Antimicrobial susceptibility testing revealed that the isolate donor strain B5 exhibited extensive drug resistance to all tested antibiotics except tigecycline and colistin (Table 1). In contrast, the recipient strain J53 demonstrated full susceptibility to the same antimicrobial panel. The J53 (pB5-KPC-NDM) displayed significantly elevated MIC values compared to J53, confirming the successful horizontal transfer of a plasmid carrying resistance genes, including *bla*_KPC-2_ and *bla*_NDM-13_. While B5 and J53 (pB5-KPC-NDM) shared nearly identical resistance profiles, differential susceptibility to levofloxacin was observed between the two isolates. B5 was resistant to levofloxacin (MIC > 8 mg/L), whereas the J53 (pB5-KPC-NDM) was susceptible (MIC ≤ 1 mg/L), and so is J53.

**TABLE 1:** Antimicrobial susceptibilities of isolates in this study (mg/L)^*a*^

Isolates	AMC	TZP	CXM	FOX	CAZ	CRO	FEP	ETP	IPM	MEM	AMK	LVX	TGC	CST
B5	>32/16	>64/4	>16	>16	>32	>32	>16	>2	>8	>8	>32	>8	≤1	≤1
J53 (pB5-KPC-NDM)	>32/16	>64/4	>16	>16	>32	>32	>16	>2	>8	>8	>32	≤1	≤1	≤1
J53	≤8	≤4/4	≤4	≤4	≤1	≤1	≤1	≤0.25	0.5	≤0.125	≤8	≤1	≤1	≤1

^*a*^ AMC, amoxicillin-clavulanate; TZP, piperacillin/tazobactam; CXM, cefuroxime; FOX, cefoxitin; CAZ, ceftazidime; CRO, ceftriaxone; FEP, cefepime; ETP, ertapenem; IPM, imipenem; MEM, meropenem; AMK, amikacin; LVX, levofloxacin; TGC, tigecycline; CST, colistin.

### Genetic features of B5

The total length of the B5 gene sequences was 1,090,782,928 bp, with an average GC content of 50.55%. WGS analysis indicated that B5 contained a 5,117,658 bp chromosome and 11 plasmids (Table 2). ANI analysis was performed between strain B5 and reference genomes of various species using FastANI software. The highest ANI value (96.79%) was observed with *E. coli* str. K-12 substr. MG1655 (NC_000913.3). MLST analysis showed that B5 belonged to ST131. Antimicrobial resistance genes were identified, including carbapenemases (*bla*_KPC-2_ and *bla*_NDM-13_), aminoglycosides (*aph(3’)-IIa* and *rmtB*), *β*-lactams (*bla*_SHV-12_, *bla*_TEM-1B_, and *bla*_CTX-M-65_), quinolones (*qnrD1*), and fosfomycin (*fosA3*) (Table 2). Notably, almost all antibiotic resistance genes, including *bla*_KPC-2_ and *bla*_NDM-13_, were located on pB5-KPC-NDM, while the *qnrD1* gene is found on plasmid5.

**TABLE 2:** Genomic characteristics of *E. coli* isolate B5

Genetic material	Accession number	Plasmid type	Size (bp)	GC content (%)	Antimicrobial resistance genes
Chromosome	CP176051	–^*a*^	5,117,658	50.64	
pB5-KPC-NDM	CP176052	IncB/O/K/Z	147,286	53.93	*bla*_KPC-2_, *bla*_NDM-13_, *aph(3’)-IIa*, *rmtB*, *bla*_SHV-12_, *bla*_TEM-1B_, *bla*_CTX-M-65_, *fosA3*
Plasmid2	CP176053	IncFIA/B/IncFII	92,949	50.88	
Plasmid3	CP176054	ColRNAI	10,060	55.06	
Plasmid4	CP176055	Col156	5,167	47.51	
Plasmid5	CP176056	Col3M	4,270	47.14	*qnrD1*
Plasmid6	CP176057	–	4,077	49.91	
Plasmid7	CP176058	ColpEC648	4,063	51.42	
Plasmid8	CP176059	–	2,953	64.75	
Plasmid9	CP176060	Col (BS512)	2,101	47.12	
Plasmid10	CP176061	Col (MG828)	1,597	49.91	
Plasmid11	CP176062	Col (MG828)	1,549	51.00	

^*a*^ "–" represents undefined plasmid replicon.

### Transferability and stability of pB5-KPC-NDM

**Fig 1:**
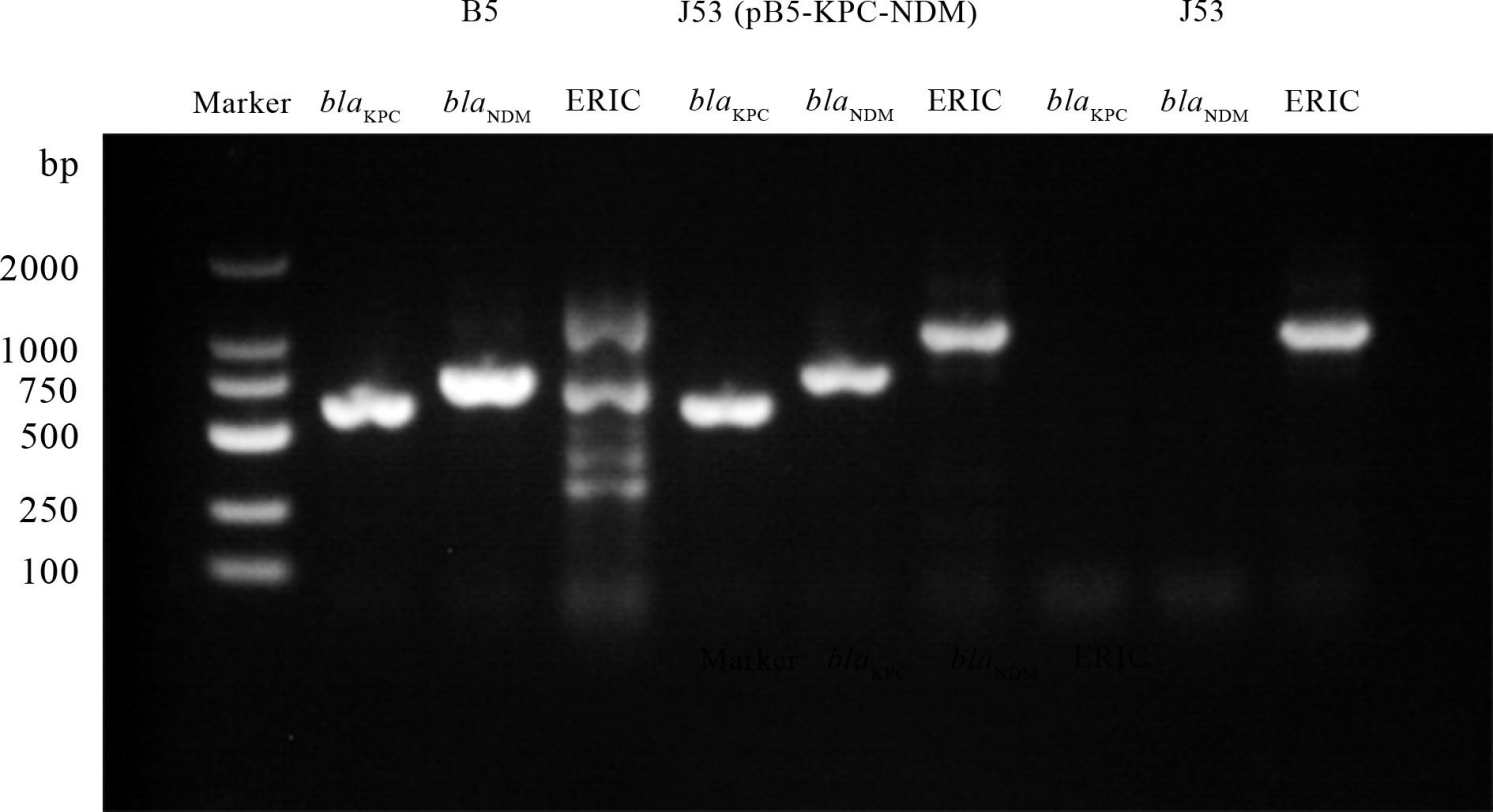
Amplifications of *bla*_KPC_, *bla*_NDM_, and ERIC sequences of *E. coli* isolate B5, J53 (pB5-KPC-NDM), and *E. coli* isolate J53.

Conjugation experiments confirmed the horizontal transfer of pB5-KPC-NDM from donor strain B5 to recipient strain J53, with *bla*_KPC-2_, *bla*_NDM-13_, and ERIC sequences of J53 detected in transconjugants (Fig. 1). Analysis of the third-generation sequencing result for the transconjugant demonstrated the presence of a complete plasmid, which was transferred to J53 with a sequence identical to pB5-KPC-NDM found in the donor strain B5. Furthermore, results from serial passaging experiments demonstrated that the stability retention rate of pB5-KPC-NDM in transconjugants was 88% (44/50) on day 5 and 74% (37/50) on day 10 in an antibiotic-free environment.

### Fitness cost of J53 (pB5-KPC-NDM)

**Fig 2:**
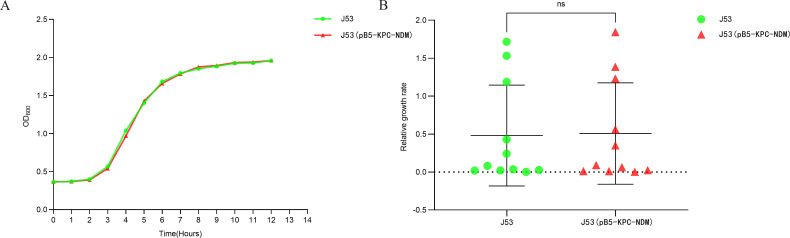
(**A**) Growth curves of J53, J53 (pB5-KPC-NDM). (**B**) Relative growth rates of J53, J53 (pB5-KPC-NDM).

J53 (pB5-KPC-NDM) exhibited no significant growth rate reduction relative to J53 (*P* = 0.972), indicating minimal fitness cost associated with acquisition of the pB5-KPC-NDM plasmid by J53 (pB5-KPC-NDM) (Fig. 2).

### Genetic characteristics of the IncB/O/K/Z plasmid pB5-KPC-NDM

**Fig 3:**
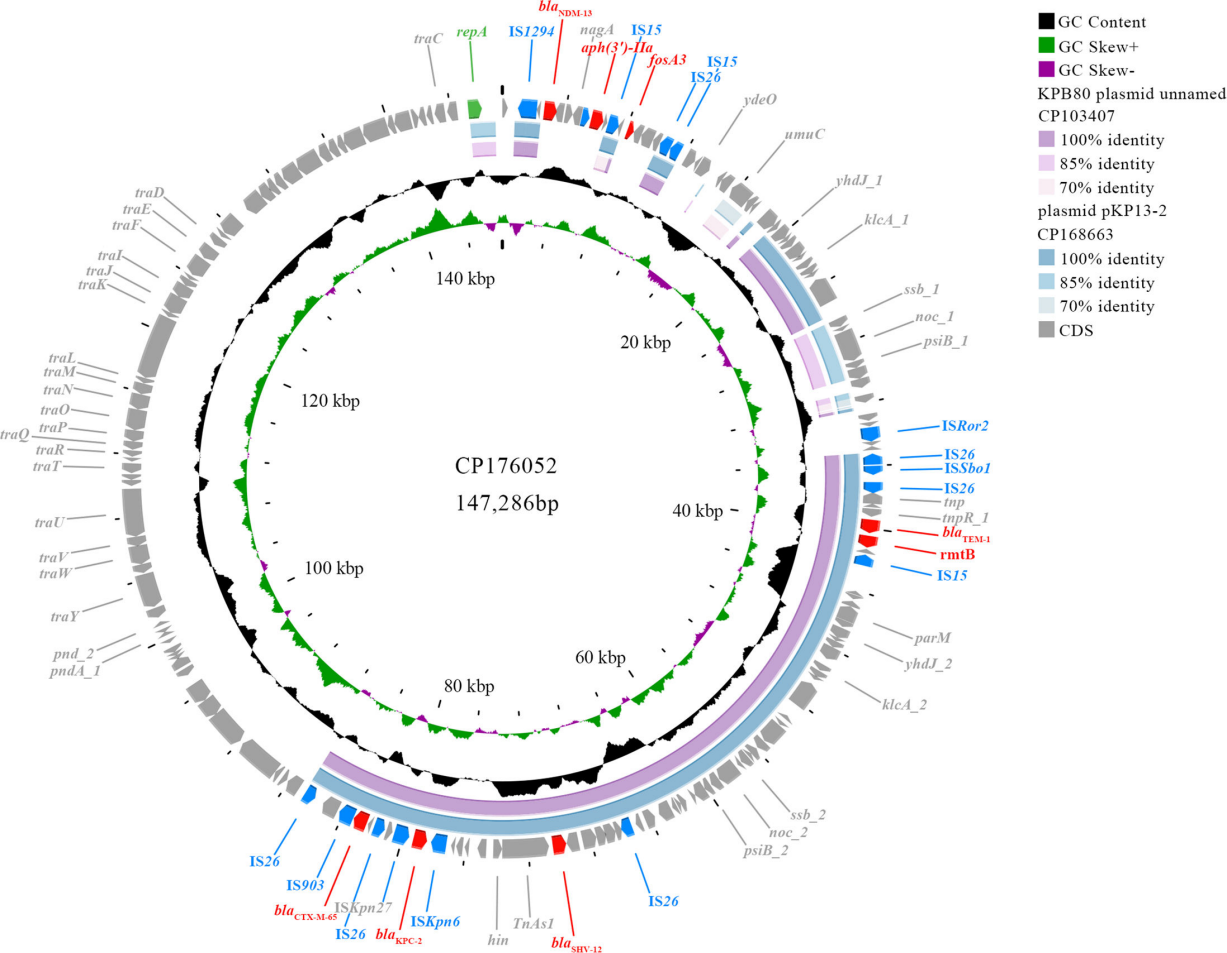
Characterization of the pB5-KPC-NDM of B5 with closely related plasmids. Plasmid structure of pB5-KPC-NDM (CP176052). pB5-KPC-NDM was used as the reference plasmid to perform genome alignment with the KPB80 plasmid unnamed2 (CP103407) and plasmid pKP13-2 (CP168663).

**Fig 4:**
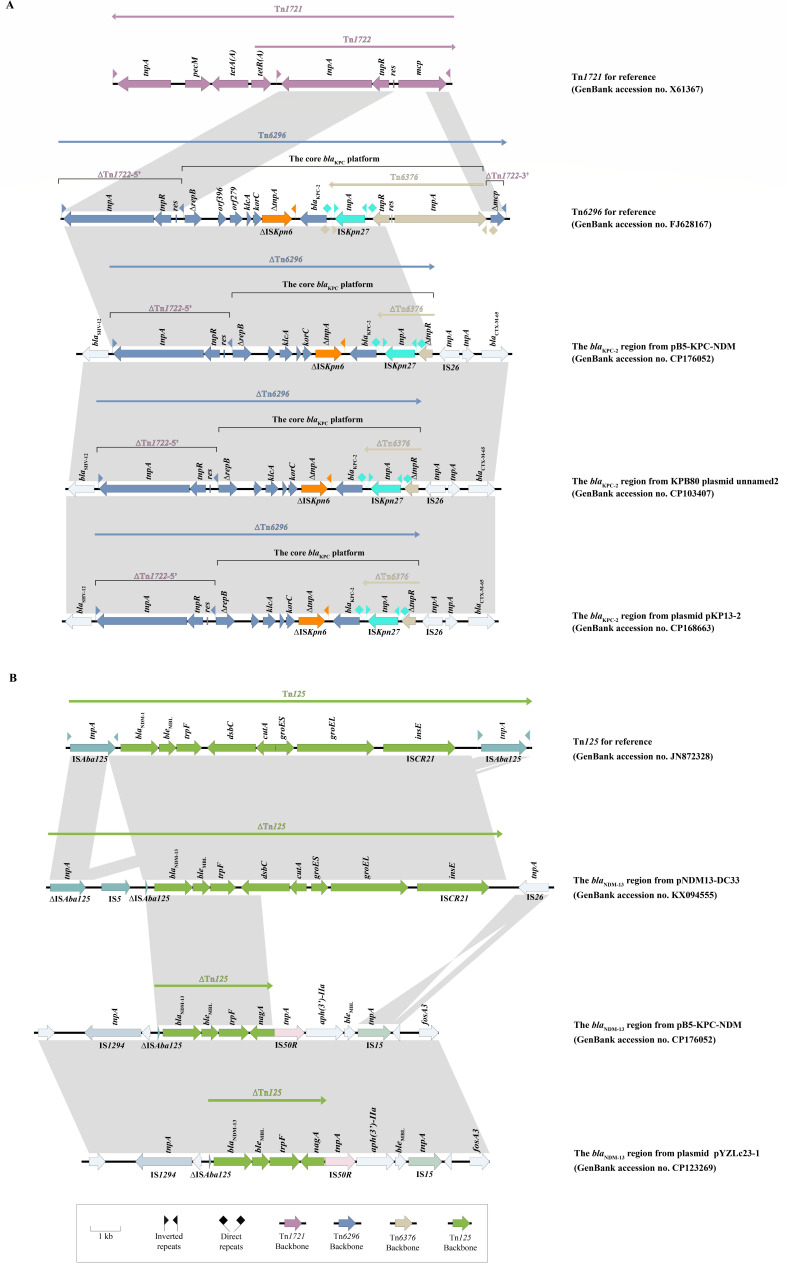
(**A**) The *bla*_KPC-2_ accessory regions in pB5-KPC-NDM, and comparison with Tn*1721*, Tn*6296*, and Tn*6376*. (**B**) The accessory *bla*_NDM-13_ regions in pB5-KPC-NDM, and comparison with Tn*125*. Genes are denoted by arrows. Genes, mobile elements, and other features are colored based on function classification. Shading denotes regions of homology (>95% nucleotide identity). The GenBank accession numbers of Tn*1721*, Tn*6296*, KPB80 plasmid unnamed2, plasmid pKP13-2, Tn*125*, pNDM13-DC33, and pYZLc23-1 are X61367, FJ628167, CP103407, CP168663, JN872328, KX094555, and CP123269, respectively.

pB5-KPC-NDM was 147,286 bp in size and had a GC content of 53.93% (Table 2). As shown in Fig. 3, pB5-KPC-NDM harbors various resistance genes alongside critical functional elements, including the replication initiator gene (*RepB*), conjugative transfer gene cluster (*Tra*), and IS elements. Comparative genomics using pB5-KPC-NDM as a reference showed 47% maximum sequence homology with related plasmids in NCBI databases, with all top 48 matches originating from *K. pneumoniae* isolates. Among these, two plasmids—plasmid pKP13-2 (CP168663) isolated from a *K. pneumoniae* in Jiangxi, and KPB80 plasmid unnamed2 (CP103407) isolated from a *K. pneumoniae* in Shanghai—exhibited 47% query coverage and 100% nucleotide identity when compared to pB5-KPC-NDM (Fig. 3). Both plasmid pKP13-2 and KPB80 plasmid unnamed2, belonging to the IncFII replicon type, carried *bla*_KPC-2_ from *K. pneumoniae* isolates that do not possess *bla*_NDM-13_. In this study, the *bla*_KPC-2_ region of pB5-KPC-NDM is approximately 10.5 kb in size and consists of a derivative of Tn*6296*, which was the same as the *bla*_KPC-2_ genetic environment section of plasmid pKP13-2 and KPB80 plasmid unnamed2 (Fig. 4A). Tn*6296* arose through insertion of the core *bla*_KPC-2_ genetic platform (Tn*6376-bla*_KPC-2_-IS*Kpn6-korC-klcA-repB*) into Tn*1722*, resulting in truncation of the *mcp* gene. This insertion truncated *mcp* and split Tn*1722* into two segments: Tn*1722*-5′ (containing the left inverted repeat, *tnpA*, *tnpR*, and *res*) and Tn*1722*-3′ (containing the truncated *mcp* and the right inverted repeat). In this study, the *bla*_KPC-2_ gene in pB5-KPC-NDM was flanked by ∆Tn*6376* (containing *tnpR*-IS*Kpn27*) and ∆IS*Kpn6* (a member of the IS*1182* family, which was originally 1,540 bp long but reduced to 981 bp), and was followed by the *korC-klcA-∆repB* genes. Comparison of the *bla*_KPC_ region in pB5-KPC-NDM with the full Tn*6296* sequence revealed that the 558 bp *tnpR* gene of Tn*6376* in pB5-KPC-NDM was truncated to 402 bp. Additionally, the ∆Tn*6376*-5′ (*tnpA-res*) and ∆Tn*1722*-3′ regions were deleted and replaced by IS*26*. Other *β*-lactamase resistance genes, including *bla*_SHV-12_ and *bla*_CTX-M-65_, coexisted around the *bla*_KPC-2_ of pB5-KPC-NDM and the compared plasmids (plasmid pKP13-2 and KPB80 plasmid unnamed2).

In addition, further analysis showed that the surrounding genetic environment of *bla*_NDM-13_ carried by pB5-KPC-NDM partially matched a previously reported *bla*_NDM-13_-harboring plasmid from pYZLc23-1 (75% query coverage and 96.4% nucleotide identity, CP123269). The genetic environment of *bla*_NDM-13_ was revealed with a 4,041 bp conserved gene arrangement of IS*1294*-∆IS*Aba125-bla*_NDM-13_-*ble*_MBL_-*trpF-nagA*, which was also detected in pYZLc23-1 (Fig. 4B). The IS*1294* was located upstream of *bla*_NDM-13_, and *ble*_MBL_ was located downstream of *bla*_NDM-13_. The genes *trpF* (encoding a phosphoribosyl anthranilate isomerase) and *nagA* (coding for the enzymes of N-acetylglucosamine uptake and metabolism) were present. The complete Tn*125* structure was flanked by two IS*Aba125* elements, with a central region consisting of *bla*_NDM-1_-*ble*_MBL_-*trpF-dsbC-cutA-groES-groEL*-IS*CR21*. Analysis of the first reported complete sequence of the *bla*_NDM-13_-harboring plasmid (pNDM13-DC33) revealed that all gene structures of Tn*125* were present in the vicinity of *bla*_NDM-13_, with the distinction that the truncated IS*Aba125* was distributed on one side of *bla*_NDM-13_. In comparison to the complete Tn*125* structure, pB5-KPC-NDM retained a partial Tn*125* structure (*bla*_NDM-13_-*ble*_MBL_-*trpF-nagA*), with a truncated IS*Aba125* upstream of *bla*_NDM-13_. This ∆IS*Aba125* shared 95% homology with the truncated IS*Aba125* in pNDM13-DC33. Notably, in the present study, IS*1294* replaced IS*Aba125* and was located upstream of *bla*_NDM-13_, differing from the genetic context of *bla*_NDM-13_ in pNDM13-DC33.

## DISCUSSION

It is extremely concerning when antimicrobial resistance genes spread via mobile genetic elements, and one of the most crucial ways is through the horizontal transfer of plasmids (18). WGS analysis of the B5 isolate revealed distinct genetic features compared to previously reported *bla*_KPC-2_ or *bla*_NDM-13_ carrying isolates. These variations are primarily attributed to distinct genomic architectures among isolates, particularly the differential arrangements of mobile genetic elements flanking resistance determinants and associated ISs within their chromosomal contexts (19–21). Notably, pB5-KPC-NDM co-harboring *bla*_KPC-2_ and *bla*_NDM-13_ maintained stability in B5 despite these genetic background variations. To our knowledge, this study provides the first evidence of *bla*_KPC-2_ and *bla*_NDM-13_ co-occurring on an IncB/O/K/Z plasmid, as well as the first detection of these genes in a clinical *E. coli* isolate (B5).

According to the antimicrobial susceptibility test results, B5 exhibited extensive drug resistance to nearly all commonly used antibiotics except tigecycline and colistin. Due to the simultaneous production of *bla*_KPC-2_ and *bla*_NDM-13_, neither aztreonam nor any *β*-lactamase inhibitors (including avibactam) were effective against this isolate (3). Furthermore, the donor strain B5 was resistant to levofloxacin, whereas J53 (pB5-KPC-NDM) exhibited a susceptible phenotype identical to the recipient strain J53. This discrepancy finds a clear explanation in the genomic analysis. The *qnrD1* gene, which confers resistance to fluoroquinolones, was located on plasmid5 of the donor strain B5. Crucially, sequencing of the transconjugant confirmed the absence of plasmid5, indicating that it was not co-transferred with the pB5-KPC-NDM plasmid during conjugation. Consequently, the lack of *qnrD1* in J53 (pB5-KPC-NDM) accounts for its susceptible phenotype to levofloxacin. The sensitivity of this isolate to colistin and tigecycline suggests their potential as a therapeutic option. Nevertheless, the increasing prevalence of CRE has led to heightened reliance on colistin and tigecycline, which poses a significant risk of developing colistin and tigecycline resistance (22).

Research indicates that *bla*_NDM_-carrying *E. coli* isolates exhibit diverse MLST types without forming dominant clones, with notable diversity observed among the plasmids carrying *bla*_NDM_ (12). This suggests the polyphyletic origins of *bla*_NDM_-positive strains and recurrent horizontal acquisition of *bla*_NDM_ across bacterial species (12). Since its initial identification on the chromosome of *E. coli* from Nepal in 2015 (11), the *bla*_NDM-13_ has disseminated via multiple plasmid types (e.g., IncX3, IncFIB/IncFII) in *E. coli* (19–23). In 2003, four isolates of *K. pneumoniae* obtained from patients at a Maryland medical center were found to produce a novel class A, plasmid-mediated *bla*_KPC-2_, which shares 99% identity with *bla*_KPC-1_. Compared to *K. pneumoniae*, *bla*_KPC-2_ transmission in *E. coli* remains comparatively restricted, primarily involving IncP6, IncR, and IncC replicons (10, 24). Notably, previous studies have found that IncB/O/K/Z plasmids are recognized vectors for multidrug resistance genes (e.g., *bla*_CTX-M_ and *aadA5*) in *Enterobacteriaceae* (25–27). The *E. coli* isolate B5 described in this study co-carries an IncB/O/K/Z plasmid harboring *bla*_KPC-2_ and *bla*_NDM-13_, thereby increasing the diversity of plasmids associated with *bla*_NDM-13_.

In a study cohort comprising up to seven CRKPs co-carrying *bla*_KPC-2_ and *bla*_NDM-1_, it was demonstrated that these seven CRKPs emerged from a CRKP carrying *bla*_KPC-2_ progenitor that subsequently acquired a highly transferable *bla*_NDM-1_ plasmid (28). This phenomenon suggests that pB5-KPC-NDM from isolate B5 may represent a fusion of multiple plasmids or mutations in genes, which appears to be a common mechanism by which bacteria acquire multiple carbapenem resistance genes. Structurally, plasmids comprise two distinct functional domains: conserved and variable. The conserved region can be further subdivided into two fragments: one encoding genes related to conjugative transfer and the other encoding backbone genes associated with plasmid replication and stability. In this study, the variable region was rich in IS elements (e.g., IS*Kpn27*, IS*26*, IS*Kpn6*, IS*1294*, IS*Aba125*) and various resistance genes, including *bla*_CTX-M-65_ and *bla*_SHV-12_. Resistance genes can be moved by IS as a component of a composite transposon, which is a region that is surrounded by two identical or related IS copies that can move together (28). Transposons and ISs allow bacteria to migrate within or across DNA molecules and acquire antibiotic resistance determinants, which play a central role in promoting the acquisition and spread of resistance genes (28). Research indicates that the *bla*_KPC_ gene is associated with diverse mobile genetic elements on transferable plasmids (13, 29). Internationally, *bla*_KPC_ typically resides within Tn*4401*, which mediates its horizontal transfer (13). The elements Tn*3-tnpR*, Tn*3-tnpA*, IS*Kpn7*, *bla*_KPC_, and IS*Kpn6* in the *bla*_KPC-2_ region of pB5-KPC-NDM are all components of the Tn*4401* isoform (29). In contrast, a distinct genetic environment for *bla*_KPC_ predominates in China, where nearly all *bla*_KPC_ genes localize to Tn*3*-Tn*4401* chimeras. These chimeras may insert into the transposon Tn*1721*. A recent study discovered that an isolate of *K. pneumoniae* co-carried *bla*_KPC-2_ and *bla*_NDM-13_, exhibiting a *bla*_KPC-2_ environment on an IncFII/IncR plasmid that was consistent with pB5-KPC-NDM (13). The IncFII/IncR plasmid possessed essential structural characteristics similar to Tn*4401* and the Tn*1721*-based structure, which are two of the most prevalent genetic environments harboring *bla*_KPC-2_ in China, the US, and Europe (13). Furthermore, Tn*6296*—a complex transposon formed by the core *bla*_KPC_ platform inserted into Tn*1722* and co-existing with Tn*1721*—serves as a key element in the dissemination of *bla*_KPC-2_ in pB5-KPC-NDM. The replacement of missing regions ∆Tn*6376*-5′ (*tnpA-res*) and ∆Tn*1722*-3′ (*mcp*) by IS*26* in pB5-KPC-NDM suggests its potential involvement in *bla*_KPC-2_ horizontal dissemination.

Tn*125*, a composite transposon flanked by two IS*Aba125* elements, facilitates the inter-species transfer of *bla*_NDM_ among bacteria (30). As a member of the IS*30* family, IS*Aba125* encodes a 322-amino-acid DDE-type transposase and enhances carbapenem resistance by promoting *bla*_NDM-1_ expression and transmission (31). Among *Enterobacteriaceae*, the ISs flanking *bla*_NDM_ (both upstream and downstream) are diverse, including IS*3000*, IS*26*, IS*EC33*, and IS*CR1*. These ISs are often deleted or truncated by other ISs, leading to structural variations (30). Despite differences in the ISs that mediate *bla*_NDM-1_ transfer, the partial Tn*125* structure (IS*Aba125-bla*_NDM-1_-*ble*_MBL_-*trpF*) remains relatively conserved, as does pB5-KPC-NDM (18, 32). Compared to previous reports (19, 20), IS*1294*, which was located in the *bla*_NDM-13_ conserved genetic sequence in pB5-KPC-NDM of B5, replaces IS*Aba125*. IS*1294*, which mobilizes via rolling circle replication, participated in the movement of *bla*_CMY-2_ (18). Interestingly, this study identifies IS*1294*, a member of the IS*91* family, which may supplant IS*Aba125* in *bla*_NDM-13_ mobilization and propagation, though *bla*_NDM-13_ remains underrepresented in surveillance data due to its historical plasmid rarity (32). Recent reports of *bla*_NDM-13_ dissemination across species underscore its emerging epidemic potential (13, 19, 20). Consequently, plasmids harboring *bla*_NDM-13_ may eventually become widespread through evolutionary processes, and urgent measures must be taken to prevent the further spread of such plasmids.

In addition, the growth rate determination results indicate that pB5-KPC-NDM incurs low fitness costs to the recipient isolate. While a previous study reported a 72% stability rate for *bla*_KPC-2_ on the tenth day (21), the similarly noteworthy stability observed in this study (pB5-KPC-NDM) establishes favorable conditions for the dissemination of carbapenem resistance genes. However, only pB5-KPC-NDM was experimentally confirmed in this study for its presence, conjugative transfer, and genetic stability. The nature of the other 10 putative plasmids remains undetermined. The convergence of *bla*_KPC-2_ and *bla*_NDM-13_ on a transmissible IncB/O/K/Z plasmid constitutes a critical public health threat, mandating intensified surveillance and containment strategies.

### Conclusion

This study characterizes a novel IncB/O/K/Z plasmid co-harboring *bla*_KPC-2_ and *bla*_NDM-13_, demonstrating exceptional conjugation stability in clinical *E. coli* isolates. Genomic analyses revealed that *bla*_KPC-2_ and *bla*_NDM-13_ are embedded within mobile genetic architectures containing multiple IS elements, suggesting potential mechanisms for horizontal gene transfer. Notably, this represents the first documentation of *bla*_KPC-2_ and *bla*_NDM-13_ co-localization on a single plasmid within the IncB/O/K/Z incompatibility group, expanding the known diversity of plasmids carrying *bla*_KPC-2_ and *bla*_NDM-13_. In addition, the genomic plasticity observed in this hybrid plasmid poses significant clinical challenges. Further research on isolates concurrently harboring multiple carbapenemases is urgently needed, given that these groups may exacerbate the spread of resistance genes and restrict antibiotic treatment options. Implementation of enhanced surveillance protocols by healthcare institutions and regulatory agencies, coupled with the development of targeted intervention strategies, is urgently required to mitigate this emerging resistance crisis.

## Data Availability

The genome sequences of isolate B5 and its 11 plasmids mentioned in the present study were submitted to the NCBI GenBank database with accession numbers CP176051 (chromosome), CP176052 (pB5-KPC-NDM), and CP176053–CP176062 (plasmid 2 to plasmid 11).
